# Implementation of coordinated spontaneous awakening and breathing trials using *t*elehealth-*e*nabled, real-time *a*udit and feedback for *c*linician ad*h*erence (TEACH): a type II hybrid effectiveness-implementation cluster-randomized trial

**DOI:** 10.1186/s13012-023-01303-1

**Published:** 2023-09-21

**Authors:** Colin K. Grissom, Richard Holubkov, Lori Carpenter, Bridgett Hanna, Jason R. Jacobs, Christopher Jones, Andrew J. Knighton, Lindsay Leither, Dee Lisonbee, Ithan D. Peltan, Carrie Winberg, Doug Wolfe, Rajendu Srivastava

**Affiliations:** 1 Department of Pulmonary and Critical Care Medicine, Intermountain Medical Center, Murray, UT 84107 USA; 2 Division of Pulmonary and Critical Care, Department of Medicine, University of Utah, Salt Lake City, UT USA; 3 Critical Care Operations, Intermountain Health, Canyons Region, Murray, UT USA; 4 Division of Pediatric Critical Care, Department of Pediatrics, University of Utah, Salt Lake City, UT USA; 5 Respiratory Care, Intermountain Health, Canyons Region, Salt Lake City, UT USA; 6 Healthcare Delivery Institute, Intermountain Health, Salt Lake City, UT USA; 7 Division of Pediatric Hospital Medicine, Department of Pediatrics, University of Utah and Primary Children’s Hospital, Salt Lake City, UT USA

**Keywords:** Mechanical ventilation, Spontaneous awakening trials, Spontaneous breathing trials, Telehealth, Audit and feedback, Implementation, Hybrid effectiveness-implementation trials

## Abstract

**Background:**

Intensive care unit (ICU) patients on mechanical ventilation often require sedation and analgesia to improve comfort and decrease pain. Prolonged sedation and analgesia, however, may increase time on mechanical ventilation, risk for ventilator associated pneumonia, and delirium. Coordinated interruptions in sedation [spontaneous awakening trials (SATs)] and spontaneous breathing trials (SBTs) increase ventilator-free days and improve mortality. Coordination of SATs and SBTs is difficult with substantial implementation barriers due to difficult-to-execute sequencing between nurses and respiratory therapists. Telehealth-enabled remote care has the potential to overcome these barriers and improve coordinated SAT and SBT adherence by enabling proactive high-risk patient monitoring, surveillance, and real-time assistance to frontline ICU teams.

**Methods:**

The *t*elehealth-*e*nabled, real-time *a*udit and feedback for *c*linician ad*h*erence (TEACH) study will determine whether adding a telehealth augmented real-time audit and feedback to a usual supervisor-led audit and feedback intervention will yield higher coordinated SAT and SBT adherence and more ventilator-free days in mechanically ventilated patients than a usual supervisor-led audit and feedback intervention alone in a type II hybrid effectiveness-implementation cluster-randomized clinical trial in 12 Intermountain Health hospitals with 15 ICUs. In the active comparator control group (six hospitals), the only intervention is the usual supervisor-led audit and feedback implementation. The telehealth-enabled support (TEACH) intervention in six hospitals adds real-time identification of patients eligible for a coordinated SAT and SBT and consultative input from telehealth respiratory therapists, nurses, and physicians to the bedside clinicians to promote adherence including real-time assistance with execution. All intubated and mechanically ventilated patients ≥ 16 years of age are eligible for enrollment except for patients who die on the day of intubation or have preexisting brain death. Based on preliminary power analyses, we plan a 36-month intervention period that includes a 90-day run-in period. Estimated enrollment in the final analysis is up to 9900 mechanically ventilated patients over 33 months.

**Discussion:**

The TEACH study will enhance implementation science by providing insight into how a telehealth intervention augmenting a usual audit and feedback implementation may improve adherence to coordinated SAT and SBT and increase ventilator-free days.

**Trial registration:**

Clinicaltrials.gov, NCT05141396, registered 12/02/2021.

**Supplementary Information:**

The online version contains supplementary material available at 10.1186/s13012-023-01303-1.

Contributions to the literature

- Implementation of effective interventions to coordinate spontaneous awakening trials and spontaneous breathing trials (C-SAT/SBT) for mechanically ventilated adults is essential to improve patient outcomes and minimize harm and inefficiencies.
- Prior studies demonstrate a need for more effective implementation strategies that are embraced by front-line clinicians in intensive care units.
- The TEACH clinical trial will be the first to evaluate a telehealth intervention augmenting common implementation strategies (audit and feedback), using a rigorous multicenter type II hybrid effectiveness-implementation cluster randomized trial design, and producing new knowledge to inform if telehealth can be a more acceptable intervention to clinicians.

## Background

Although invasive mechanical ventilation is a lifesaving treatment for more than 400,000 US patients with acute respiratory failure each year [1], it is associated with significant risk of preventable harm. Sedation and analgesia improve patient comfort and synchrony with the mechanical ventilator, reduce oxygen consumption, and decrease pain. Prolonged sedation, however, may increase time on mechanical ventilation and risk for ventilator associated pneumonia, delirium, and poor long-term cognitive outcomes. The benefits of sedation and analgesia need to be balanced by the potential harm of prolonged sedation and mechanical ventilation.

Spontaneous awakening and breathing trials during mechanical ventilation improve patient outcomes. Daily interruptions in sedation [spontaneous awakening trials (SATs)] decrease duration of mechanical ventilation and intensive care unit (ICU) length of stay without compromising patient comfort or safety [2–4]. Daily spontaneous breathing trials (SBTs) decrease duration of mechanical ventilation and reduce costs in ICU patients regardless of the etiology of respiratory failure [5–10]. Coordinated SATs and SBTs (C-SAT/SBTs) — whereby the SAT is followed by an SBT within a short window of time — increase ventilator-free days, decrease ICU length of stay, improve mortality [11], and reduce ventilator-associated events [12]. Implemented as part of a coordinated bundle, C-SAT/SBT improves mortality and reduces ICU costs [13–15].

Spontaneous awakening and breathing trials are difficult to coordinate and face substantial implementation barriers. Initial SAT/SBT trials utilized relatively narrow inclusion/exclusion criteria and employed resource-intensive methods for ensuring high adherence and careful monitoring of strictly protocolized C-SAT/SBT. Subsequent implementation efforts have focused on multistep protocols driven by nurses who conduct SATs and respiratory therapists who conduct SBTs [3, 5, 11, 15–19]. Despite these implementation efforts and inclusion of daily C-SAT/SBT in guidelines [20–22], adherence remains low [23, 24]. Published adherence rates range from 54 to 96% for SAT [25–30] and 29 to 97% for SBT [12, 31–37]. Variable adherence persists even when C-SAT/SBT is included in awakening, breathing coordination, delirium monitoring/management, and early exercise/mobility bundles. There is a critical need to identify best practices for overcoming barriers to C-SAT/SBT use [24].

C-SAT/SBT implementation requires a clear understanding of protocol steps and tight coordination of difficult-to-execute sequencing between nurses and respiratory therapists [23, 24, 30, 35]. Barriers specific to C-SAT/SBT implementation include the following: (1) patient: clinical stability and safety; (2) clinician: lack of knowledge and awareness; (3) protocol: unclear and cumbersome; and (4) ICU system: substandard interprofessional communication and coordination [24]. Barriers common to implementation of ICU clinical practice guidelines are also important, including inadequate clinician education, dependence on clinician recall for process activation rather than decision support tools (including alerts), and a lack of local performance audit and feedback from already busy front-line supervisors to identify opportunities for improvement including coaching individual clinicians [10, 12, 16, 27, 32, 38]. ICUs are typically not staffed with personnel dedicated to facilitating adherence or leaders trained in implementation of evidence-based care [39].

Telehealth-enabled remote care, and specifically tele-critical care, has the potential to overcome these barriers and improve C-SAT/SBT use. Tele-critical care enables proactive high-risk patient monitoring, surveillance, and real-time assistance — through central audit and feedback — to front-line ICU teams [40, 41], augmenting the monitoring and prompting responsibilities placed on frontline supervisors. Tele-critical care reduces mortality, decreases length of stay, improves best practice adherence, and lowers rates of preventable ICU complications [42].

Emerging literature suggests remote monitoring and prompting to improve C-SAT/SBT are feasible and effective. One pre/post study deployed a web-based electronic dashboard with information on SBT readiness, depth of sedation, and sedative infusions. A text message alert system notified respiratory therapists and nurses of patient readiness for SAT and SBT. After implementation, mechanical ventilation days and ICU length of stay decreased by 2.2 and 2.7 days respectively [43]. However, prior published experience is limited to observational, single-center, and/or pre/post designs, all of which limit generalizability and validity of causal inference. How best to implement C-SAT/SBT using remote monitoring and prompting therefore remains a critical knowledge gap.

We designed a cluster-randomized type II hybrid effectiveness-implementation clinical trial to determine whether adding *t*elehealth-*e*nabled, real-time *a*udit and feedback for *c*linician ad*h*erence (TEACH) to a usual supervisor-led audit and feedback intervention will yield higher C-SAT/SBT adherence and more ventilator-free days in adult mechanically ventilated patients than a usual supervisor-led audit and feedback intervention alone.

## Methods/design

This manuscript adheres to the Standard Protocol Items: Recommendations for Interventional Trials (SPIRIT) [44] and the CONSORT extension for cluster-randomized trials [45] (Additional Files 1 and 2).

### Trial management and protection of human subjects

The trial is led by three principal investigators, C. K. G., R. S., and R. H., in a co-principal investigator management plan with a Coordinating Council that includes co-investigators L. L., I. D. P., A. J. K., C. W., and D.W. The Coordinating Council oversees the clinical effectiveness core, implementation science core, and the data and statistical core. The Coordinating Council has monthly or bimonthly meetings with two representatives from the National Institutes of Health, National Heart, Lung, and Blood Institute: Mihaela Stefan, program officer, and Karen Bienstock, clinical trials specialist. The Data Coordinating Center (DCC) is housed at the University of Utah under the direction of RH. A Data Safety and Monitoring Board (DSMB) has been convened (charter in Additional File 3).

This study protocol was approved by the Intermountain Health Institutional Review Board with waiver of informed consent on 7/28/2022 (no. 1051681).

### Study aim and hypotheses

The aim of this study is to determine whether adding a telehealth-enabled real-time audit and feedback intervention (TEACH) to a usual supervisor-led audit and feedback intervention yields higher adherence to C-SAT/SBT and more ventilator-free days in adult mechanically ventilated patients compared to a usual supervisor-led audit and feedback intervention alone. Along with routine supervisor orientation and training on staff development, all sites will receive the baseline implementation strategies of a standardized C-SAT/SBT clinical workflow measurement system and supporting technology (audit and feedback implementation).

The study hypothesis is that at hospitals assigned to the TEACH intervention plus usual supervisor-led audit and feedback, improvement from baseline in adherence to C-SAT/SBT and patient ventilator-free days will be higher than that observed at hospitals assigned to usual supervisor-led audit and feedback alone.

### Trial overview and design

**Fig. 1:**
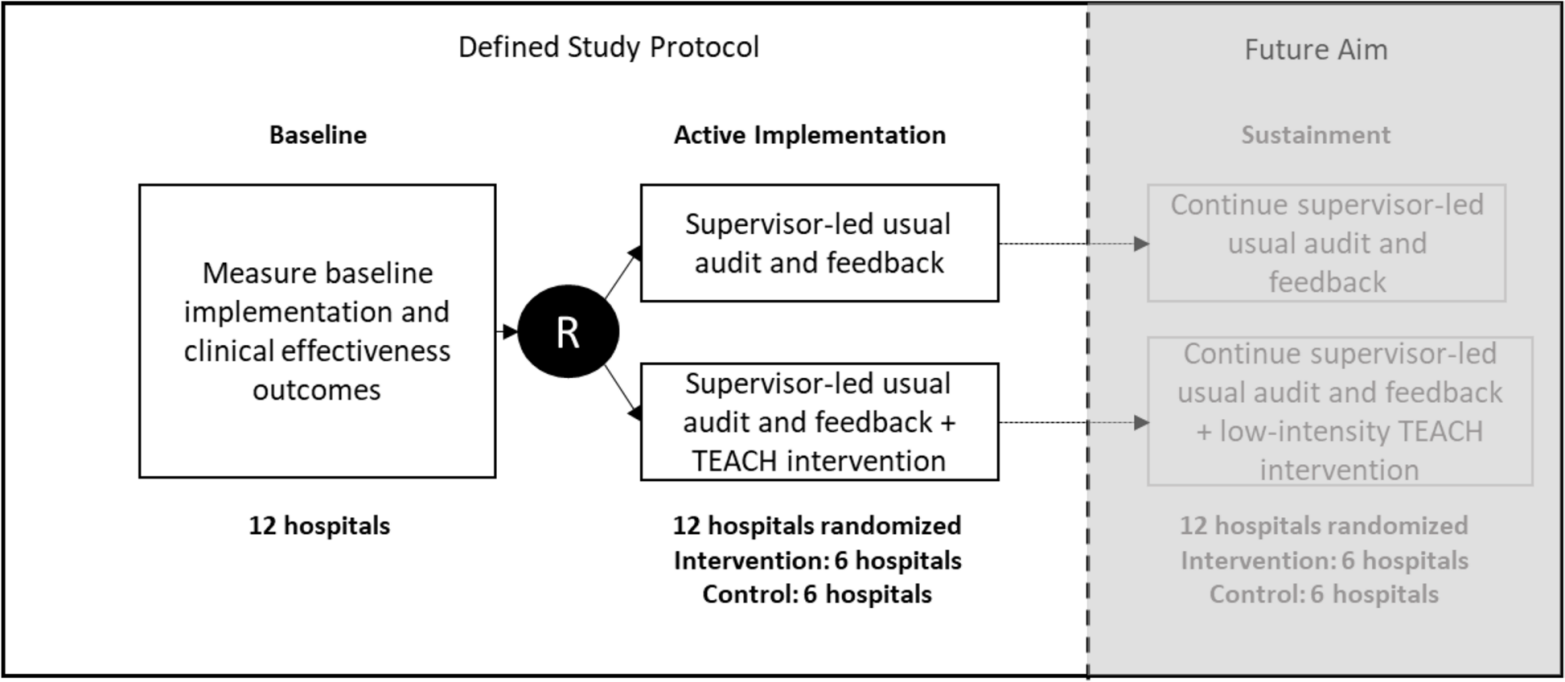
Clinical trial diagram showing the measurement of baseline implementation and clinical effectiveness outcomes, randomization (R) of the 12 study hospitals to supervisor-led audit and feedback (6 hospitals) or supervisor-led audit and feedback plus the TEACH intervention (6 hospitals). The diagram also shows a future aim at the conclusion of the TEACH clinical trial that will evaluate sustainment in outcomes utilizing fewer resources in both the TEACH intervention and control arms

As shown in Fig. 1, this study is a prospective, multicenter type II hybrid effectiveness-implementation cluster-randomized trial comparing a usual supervisor-led audit and feedback intervention to promote uptake of C-SAT/SBT versus the usual supervisor-led audit and feedback intervention augmented with the TEACH audit and feedback intervention. The incremental TEACH audit and feedback intervention has five daily components: (1) identification of patients who may be eligible for C-SAT/SBT; (2) evaluation to confirm C-SAT/SBT eligibility; (3) outreach to bedside nurses and respiratory therapists whose patients are not charted as adherent to SAT or SBT; (4) consultative input from tele-critical care respiratory therapists, nurses, and physicians to the bedside clinicians to promote adherence, including tele-critical care real-time assistance with execution of C-SAT/SBT by bedside nurses and respiratory therapists; and (5) periodic reports to facility managers on patterns in TEACH interactions and feedback.

Type II effectiveness-implementation trials place equal importance on clinical effectiveness and implementation outcomes, an appropriate design given this study’s purpose to determine the utility of the TEACH implementation intervention as a method for (1) improving adherence to C-SAT/SBT and (2) improving clinical outcomes as measured by ventilator-free days [46, 47].

A future aim will include a sustainment period that will test if lower intensity interventions impact adherence to the implementation outcomes.

### Setting

This study will be performed in 12 hospitals with 15 ICUs serving ~ 3600 mechanically ventilated patients annually from Utah and Idaho in urban, suburban, and rural settings of Intermountain Health (Intermountain).

Intermountain operates a mature tele-critical care program, with all study ICU rooms remotely monitored by a centralized tele-critical care hub using a shared electronic medical record, networked telemetry data, and equipment for two-way audiovisual communication between clinical personnel based at the tele-critical care hub and patients and bedside providers. The tele-critical care team — comprised of experienced ICU nurses, pharmacists, critical care physicians, advanced practice providers, and respiratory therapists — co-manages patients at seven community hospital ICUs and are available for consultation on patients located in eight referral center ICUs. Intermountain has a single electronic medical record for all inpatient care in study hospitals, so the tele-critical care providers have access to the same set of clinical information as bedside providers. Tele-critical care personnel have access to an electronic dashboard that displays ventilator mode, settings, and parameters and C-SAT/SBT status for every mechanically ventilated patient across the system. Real-time tele-critical care support is ideally suited to identify C-SAT/SBT candidates, motivate C-SAT/SBT performance, and provide bedside clinical teams with expert peer-to-peer consultation regarding how to perform C-SAT/SBT accurately and act on results.

### Randomization and treatment assignment

The unit of randomization will be the hospital for this cluster-randomized clinical trial. The 12 study hospitals will be assigned by block randomization, stratified on baseline ICU-ventilated patient volume, to receive either the usual supervisor-led intervention alone (6 hospitals) or the TEACH intervention in addition to the usual supervisor-led implementation approach (6 hospitals). Block randomization by hospital and ventilated patient volumes ensures balance between treatment arms. All hospitals have one ICU except for the largest hospital, Intermountain Medical Center, with four ICUs. Since individual ICUs at this hospital share respiratory therapist, nurse, and physician staffing, all ICUs were randomized as one cluster and analyzed together to prevent between-arm contamination. Stratified randomization to TEACH versus usual implementation approach will occur in three steps: (1) randomize the two largest volume sites according to baseline ICU-ventilated patient volume, one site to usual implementation alone and one to usual implementation plus TEACH, (2) randomize the next two largest-volume sites in the same 1:1 fashion, and (3) randomize the remaining eight sites with four sites randomized to usual supervisor-led audit and feedback alone and four randomized to TEACH plus usual supervisor-led audit and feedback.

### Inclusion and exclusion criteria

All intubated and mechanically ventilated patients will be eligible for enrollment if they meet all inclusion criteria (age ≥ 16 years, admission to a study hospital ICU, and intubation and mechanical ventilation) and have no exclusion criteria (preexisting brain death with admission to study hospital for organ donation or death on the day of intubation.)

### Study procedures — usual care

In preparation for the randomized clinical trial, we will measure baseline implementation and effectiveness primary outcomes for 6 months. Results will be used as the baseline performance on the primary outcomes prior to randomization in order to perform a final power analysis and refine the final statistical analysis plan.

**Fig. 2:**
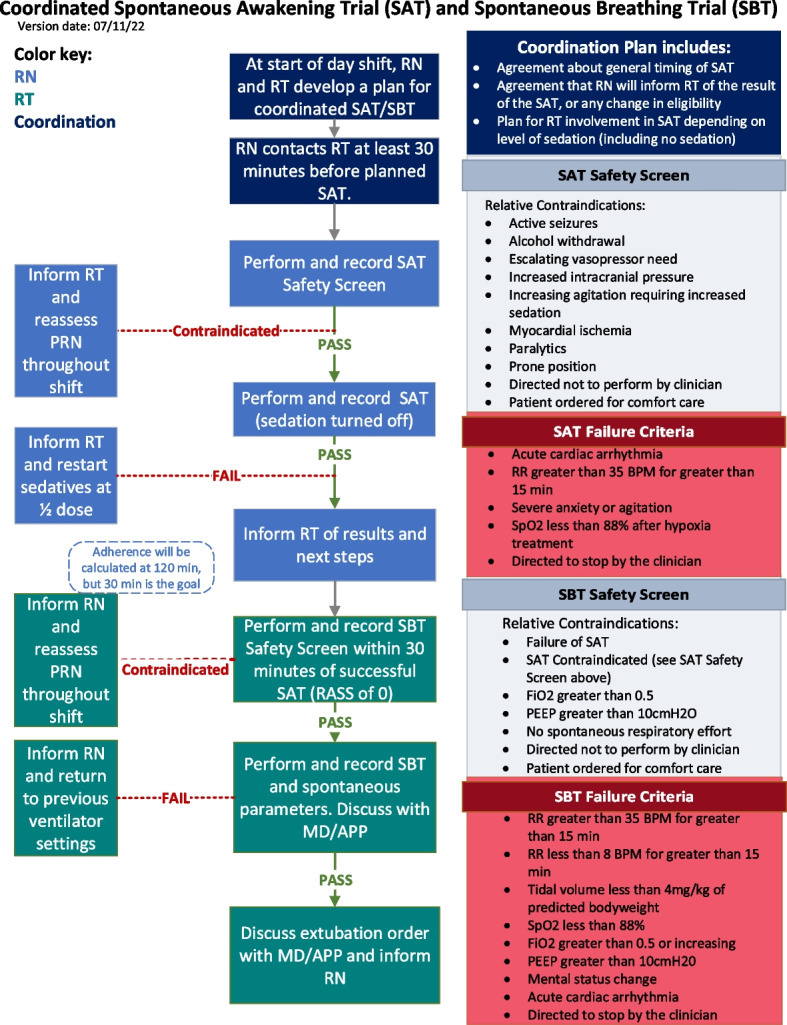
Algorithm for coordination of spontaneous awakening trials and spontaneous breathing trials color coded for nurse (light blue, RN), respiratory therapist (green, RT), and joint tasks (dark blue) including safety screens and failure criteria. MD is physician and APP is advanced practice provider

We developed a standardized C-SAT/SBT screening and performance protocol algorithm (Fig. 2) based on literature review; input from subject matter experts; input from clinicians, nurses, and respiratory therapists; and qualitative investigation of bedside practices and barriers related to C-SAT/SBT. Additional documents developed to support C-SAT/SBT include nurse and respiratory therapist job aids (Additional Files 4 and 5).

All patients will have analgesia and sedation managed according to the Intermountain “Pain, Agitation, and Delirium Guideline for Mechanically Ventilated Patients” (Additional File 6) that provides guidance for standardized, evidence-based analgesia and sedation. Agitation and delirium will be measured per standard clinical practice using the Richmond Agitation and Sedation Scale [48] to assess sedation and target a score of 0 (range − 5 to + 4) and the Confusion Assessment Method for the ICU (CAM-ICU) [49] to assess for delirium.

All patients on mechanical ventilation will be managed using clinical decision support with computerized open-loop protocols within our Cerner electronic medical record that provide instructions for ventilation, oxygenation, weaning assessment, and continuous positive airway pressure (CPAP) or pressure support (PS) weaning [50]. The ventilation protocol utilizes low tidal volume lung protective ventilation in the volume control or pressure-regulated volume control mode and normal or high positive end-expiratory pressure (PEEP) titration protocols based on the Acute Respiratory Distress Syndrome Network and Prevention and Treatment of Acute Lung Injury Network protocols [51, 52]. The ventilation protocol prompts the respiratory therapist to perform a weaning assessment when the fraction of inspired oxygen (FiO_2_) is ≤ 0.5 and PEEP ≤ 10 cm H_2_O on volume control ventilation. The respiratory therapist uses the computerized weaning assessment protocol that can advance the patient to an SBT if respiratory parameters are acceptable while breathing on CPAP of 5 to 10 cm H_2_O (whatever PEEP level the patient was on prior to the weaning assessment). The respiratory therapist then performs an SBT using the CPAP and PS computerized weaning protocols starting with PS 5 cm H_2_O and PEEP 5 to 10 cm H_2_O.

#### Daily SAT and safety screen eligibility

On eligible patient days (all days on mechanical ventilation except the first calendar date of intubation), an SAT safety screen is performed if the patient is receiving a continuous infusion of sedative or analgesics. The bedside clinician may direct that an SAT not be performed for a given patient day for clinical reasons (Fig. 2), and contraindications are documented. If no contraindications exist, the SAT safety screen is passed, and the SAT is performed.

SAT performance will entail stopping all sedative and analgesic infusions, followed by a structured assessment of the patient’s ability to remain off these medications. Patients undergoing an SAT may have analgesia, and sedation restarted at 50% of the previous dose and titrated to a Richmond Agitation and Sedation Scale of 0 to − 1 if they meet any of the failure criteria in Fig. 2.

#### Daily SBT and safety screen eligibility

On eligible patient days, an SBT safety screen is performed. The bedside clinician may direct that an SBT not be performed for a given patient day for clinical reasons (Fig. 2), and contraindications are documented. If no contraindications exist, the SBT safety screen is passed, and the SBT is performed.

An SBT will be considered performed if (1) the patient has an SBT failure per criteria (Fig. 2), (2) patient has spontaneous parameters measured while breathing on CPAP 5 to 10 cm H_2_O and FiO_2_ ≤ 0.5, or (3) the respiratory therapist documents that an SBT was performed.

If no failure criteria are met acutely, the patient will have spontaneous parameters recorded including minute ventilation, tidal volume, respiratory rate, vital capacity, and maximum inspiratory pressure. The patient will then be evaluated for extubation, usually after 30 to 120 min. If no failure criteria are met, or the patient is not extubated after the standard period of time (usually up to 120 min), then they may be continued on a CPAP or PS mode utilizing the PS/CPAP computerized weaning protocol (PS up to 15-cm H_2_O, FiO_2_ up to 0.5, and PEEP up to 10-cm H_2_O). If at any time the patient meets computerized ventilator protocol criteria (an increase of FiO_2_ to > 0.5 or *PEEP* > 10-cm H_2_O or *PS* > 15-cm H_2_O), they are returned to volume control ventilation at their previous settings.

### Study procedures — usual supervisor-led audit and feedback at all sites

Usual supervisor-led audit and feedback for nurses and respiratory therapists at Intermountain includes formal and informal conversations at least quarterly between a direct supervisor and an employee to review job performance and to encourage improvement as needed, including adherence to clinical practice standards. Information on job performance is typically captured via direct observation, conversations with the employee’s peers, and performance reports. The number, content, quality, and timing of supervisor-led audit and feedback vary by supervisor. For this study, supervisors at all sites will also receive adherence reporting weekly from a systemwide electronic dashboard on the Intermountain intranet accessible to designated providers. Reports will include adherence data at the system, ICU, clinician, and component (SAT, SBT, coordination) levels and also at the individual patient encounter and patient day levels. Supervisors will be encouraged to use report data to support C-SAT/SBT uptake and to facilitate employee audit and feedback.

To further standardize the use of supervisor-led audit and feedback and promote the use of adherence reporting to encourage C-SAT/SBT adherence, education and training activities will occur at all study sites (see Table 1). Team members from the Implementation Science and Clinical Effectiveness Cores will coordinate the deployment system wide. Site efforts will be supported by a system-wide media and communications plan. Nursing and respiratory therapy operation leaders at each ICU, with support from the ICU Team members from the Implementation physician leaders, will serve as local project leaders and champions and identify additional local champions as needed. The implementation science core will provide ICU project leaders and champions with an implementation toolkit and training to assist them in guiding local deployment and adaptation. Unit leaders will also be trained on the use of iterative improvement cycles to promote unit-level practice adherence. The implementation science core will monitor ICU performance and facilitate system-wide best practice sharing forums to promote and share local learning.

**Table 1:** Strategies to promote supervisor-led audit and feedback at all sites

Domain	Organize executive implementation team	Identify and prepare local site project team/site champions	Facilitate using an implementation advisor	Develop and distribute educational materials	Facilitate relay of clinical data to clinicians
Actor(s)	Cross-disciplinary leadership including ICU clinical and implementation leaders	Local deployment by clinical teams including site physician, nurse and respiratory care leaders, and site champions	Project leader/facilitator with expertise in process improvement and implementation	Cross-disciplinary clinical operations team including system physician, nurse and respiratory care leaders, clinical education and training, information technology	Cross-disciplinary team including clinical and implementation effectiveness leadership, information technology
Action(s)	Coordinate efforts of working teams, set performance goals, monitor performance	Monitor site performance, identify and address barriers to adherence through rapid cycle Plan-Do-Study-Act experiments	Provide consultation on use of rapid-cycle Plan-Do-Study-Act, including training and best practice sharing forums	Develop and distribute training materials, including implementation toolkit (goals, roles, contact list, protocol reference guide, FAQ)	Develop and deploy standardized C-SAT/SBT clinical workflow measurement system and supporting technology
Targets of the action	Local site project teams/champions	Front-line clinicians	Enterprise executive implementation team	Local site project teams/champions, front-line clinicians	System leadership, local site project teams/champions, front-line clinicians
Temporality	Initiation through post-implementation phase	Implementation phase	Initiation through implementation phase	Implementation phase	Initiation through post-implementation phase
Dose	At least monthly	Weekly or biweekly	Weekly/biweekly sessions as needed by site	At site deployment and as needed by site	Monthly
Implementation outcomes affected	Uptake, penetration	Uptake, penetration, fidelity	Uptake, penetration	Uptake, penetration, fidelity	Uptake, penetration, fidelity

### Study procedures — incremental TEACH audit and feedback intervention

In addition to the usual supervisor-led audit and feedback, sites randomized to the TEACH intervention will receive targeted C-SAT/SBT support from the centralized tele-critical care team. This will extend the local leader’s ability to promote increased adherence to C-SAT/SBT through direct messaging, education, and feedback. To support local ICU leaders during the initial education period, the tele-critical care nurse and respiratory therapist team members will develop educational materials and conduct trainings, including how to communicate with tele-critical care when they need assistance (see Table 2). See Additional File 7 for specific guidelines for daily tele-critical care operations in support of real-time audit and feedback for TEACH after the initial education period.

**Table 2:** Strategies for deployment of the telehealth-enabled remote audit and feedback (TEACH intervention)

Domain	Organize clinical operations leadership team	Identify and prepare local site project team/site champions	Develop educational materials and conduct training	Facilitate relay of clinical data to clinicians in real time	Utilize remote monitoring and prompting to audit and deliver feedback
Actor(s)	Nurse and respiratory care leadership, tele-critical care nurse and respiratory care leadership, implementation advisors	Cross-disciplinary site teams including local site physician leader, nurse, and respiratory care project leads	Nurse and respiratory care leadership, tele-critical care nurse and respiratory care leadership, implementation advisor	Cross-disciplinary team including clinical and implementation effectiveness leadership, information technology	Tele-critical care nurses and respiratory therapists
Action(s)	Define standard workflow for the TEACH intervention Identify and address barriers to adherence	Identify and address local barriers to adherence to the TEACH intervention	Introduce TEACH intervention in staff meetings, provide individual training and reinforcement with nurses and respiratory therapists	Utilize an electronic dashboard that includes information on SAT/SBT performance along with electronic medical record and tracking data	Identify potential non-adherent patients, evaluate to confirm nonadherence, conduct outreach to bedside clinician, provide bedside consultation/facilitation, document interaction
Targets of the action	Local site project teams/champions	Front-line clinicians	Local site project teams/champions	Tele-critical care nurses and respiratory therapists	Front-line respiratory therapists and nurses
Temporality	Initiation through sustainment	Implementation through sustainment	Implementation phase	Initiation through sustainment	Implementation through sustainment
Dose	At least biweekly	Biweekly	At site deployment and periodic refresh as needed	Daily, real-time reporting	Daily, as needed
Implementation outcomes affected	Uptake, penetration	Uptake, penetration, fidelity	Uptake, penetration, fidelity	Uptake, penetration, fidelity	Uptake, penetration, fidelity

Interactions between the tele-critical care clinician and the bedside clinician are documented in the real-time electronic dashboard. Periodic reports on patterns in TEACH interactions and feedback from front-line clinicians are provided to local leaders of tele-supported sites. Local ICU leaders and champions will be coached to use this information to craft messaging, set goals, and make adjustments as needed with local bedside nurses and respiratory therapists.

### Data collection and analysis

We will collect patient data using customized clinical data entry workflows, queries of the Intermountain comprehensive electronic data warehouse, and structured manual chart review. Data on intervention acceptability will be collected via surveys implemented using the web-based Research Electronic Data Capture (REDCap) platform [53, 54].

### Primary exposure

The primary exposure will be the implementation strategy (usual audit and feedback alone [control] or supplemented by TEACH [intervention]) assigned to the study hospital where patient was located during their second intubated calendar day.

### Other independent variables

- Patient demographics: Age, sex, race/ethnicity, relative socio-economic deprivation using a modified Singh area deprivation index [55, 56], insurance type, and comorbidities
- Clinical data: Vital signs, respiratory failure etiology, APACHE IV score, laboratory results, medications (e.g., sedation and analgesic doses), and Richmond Agitation and Sedation Scale
- Ventilator data: Ventilator settings and contraindications for SAT/SBT

### Co-primary outcome (implementation)

The co-primary outcome for implementation is adherence to C-SAT/SBT (Table 3), calculated by dividing the number of adherent C-SAT/SBT days by the number of eligible patient days. An eligible patient day is defined as any day after the initial day of intubation and mechanical ventilation during which the patient remains intubated and receiving mechanical ventilation and does not meet any exclusion criteria.

**Table 3:** Implementation and clinical effectiveness outcomes

Measurement category	Primary/secondary	Proctor implementation outcomes	Measure	Description
Implementation	Primary	Adoption	Clinician adherence to C-SAT/SBT	Percentage calculated by dividing the number of adherent C-SAT/SBT days documented by the number of eligible patient days
	Secondary	Feasibility	Incremental labor cost per patient days eligible for the TEACH intervention outreach	Incremental labor costs (base salary rate/fringe, supervisor overhead, overtime) divided by the number of patient days eligible for the TEACH intervention outreach
		Fidelity	Telehealth contact rate	Percentage calculated by dividing the number of successful contacts between the tele-critical care team and frontline nurses and RTs documented divided by the number of patient days eligible for the TEACH intervention outreach
		Acceptability	Clinician satisfaction scores	Percentage reporting agree or strongly agree on a 5-point Likert scale via a clinician survey
Clinical effectiveness	Primary	N/A	Patient ventilator free days to day 28	Number of days from the time of initiating unassisted breathing to day 28 after initiation of IMV, assuming survival for at least two consecutive calendar days after initiating unassisted breathing and continued unassisted breathing to day 28
	Secondary		Reintubation rate after intentional extubation	Proportion of patients undergoing intentional extubation who require reintubation for respiratory failure within 72 h. Excludes reintubations for < 24 h for the purpose of a surgical procedure
			Unintentional extubation incidence and reintubation rate	Separate from intentional extubation. Tracks unintentional extubations and rate of reintubation with 72 h
			Ventilator-associated pneumonia rate	New antibiotic administered in an intubated patient associated with a positive respiratory culture
			Richmond Agitation and Sedation Scale	Average Richmond Agitation and Sedation Scale each calendar day over the first 7 days from intubation. Recorded every 4 h by nursing
			CAM-ICU score	Identifies the presence or absence of delirium. Recorded every 12 h by nursing
			Delirium and coma-free days	Days free of delirium and coma to day 28. Delirium-free defined by the CAM-ICU score and coma-free defined by a RASS of > − 4
			Time to first ICU activity	Defined as a Johns Hopkins Highest Level of Mobility Scale of ≥ 2
			ICU length of stay	Duration of stay in the ICU in days
			Hospital length of stay	Duration of stay in the hospital in days
			Hospital discharge disposition	Home, skilled nursing facility, long-term acute care facility, or rehabilitation facility
			Mortality — hospital, 30-day and 90-day mortality	Raw unadjusted mortality and adjusted mortality as defined in the methods

A C-SAT/SBT adherent day is defined as an eligible patient day when the patient is both SAT adherent and SBT adherent (defined below). Additionally, if an SAT was passed, then at least one of the following criteria must be met: (1) the SBT event must also follow the SAT event and occur within 2 h, or (2) the SAT and SBT events must both occur before noon and contain charted evidence that the patient was responding to commands when the SBT event occurred.

An SAT adherent day is defined as an eligible patient day when charted evidence of any one or more of the following is documented: extubation, no sedation, SAT contraindication, successfully passed SAT, performed but failed SAT, or no SAT necessary (i.e., the patient is awake and responsive).

An SBT adherent day is defined as an eligible patient day when charted evidence of any one or more of the following is documented: extubation, SBT contraindication, documentation of spontaneous parameters, or performed SBT.

### Secondary outcomes (implementation) 

Secondary implementation outcomes were categorized using Proctor’s measurement framework [47] and includes feasibility (incremental labor hours of the telehealth nurse and respiratory therapist roles), fidelity (contact frequency between telehealth roles and bedside clinical team), and acceptability (Table 3). To report acceptability, upon achieving stable adherence to the C-SAT/SBT, the research team will disseminate a survey tool using the secure REDCap data management system to capture front-line clinician impressions regarding acceptability of either study arms. The anticipated survey population will include approximately 150 ICU physicians and advanced practice clinicians (medical/surgical intensivists and hospitalists), 575 ICU nurses and nurse supervisors, and 250 respiratory therapists and respiratory care supervisors drawn from the 12 hospital sites. To measure acceptability, we will adapt the Acceptability of Intervention Measure (AIM), a previously validated 4-item measurement instrument, informed by the Unified Theory of the Acceptance and Use of Technology [57]. AIM measures approval, appeal, likability, and general willingness to accept the incremental telehealth-enabled audit and feedback implementation strategy versus supervisor-directed audit and feedback alone using an ordinal 5-point Likert scale. We have targeted a 40% response rate for analysis purposes, sufficient to capture important differences in attitudes across intervention and control participants and clinician roles.

### Co-primary outcome (effectiveness)

The co-primary outcome for effectiveness will be patient-level ventilator-free days to day 28 (Table 3), defined as the number of days from the time of initiating unassisted breathing to day 28 after initiation of mechanical ventilation, assuming survival for at least two consecutive calendar days after initiating unassisted breathing and continued unassisted breathing to day 28 [51, 58]. If a patient returns to assisted breathing and subsequently achieves unassisted breathing to day 28, ventilator-free days will be counted from the end of the last period of assisted breathing to day 28. A period of assisted breathing lasting less than 24 h for the purpose of a surgical procedure will not count against the ventilator-free days calculation. If a patient was receiving assisted breathing at day 27, ventilator-free days will be zero; if the patient dies prior to day 28, ventilator-free days will be coded as − 1. Unassisted breathing is defined as follows: (1) extubated with face mask, nasal prong oxygen, or room air, (2) T-tube breathing, (3) tracheostomy mask breathing, (4) use of CPAP or noninvasive positive pressure ventilation solely for sleep apnea management, or (5) use of a high-flow oxygen system. For secondary effectiveness outcomes, see Table 3.

### Data management

Data management and analysis activities will occur via a collaboration between the Intermountain data management team and the University of Utah DCC. Under the subcontract agreement with the DCC and consistent with prior studies, the Intermountain study team is responsible for cleaning, aggregating, and exporting all data captured at the study sites and delivering to the University of Utah DCC for analysis via a secure server.

The DCC will present relevant study data and other trial issues to the DSMB, including the statistical trial design at the initial DSMB meeting, and the finalized trial statistical analysis plan at the end of the 6-month baseline measurement period prior to initiation of the clinical trial. The DCC will also perform two interim efficacy analyses (further described below) to consider early stopping for superiority if compelling evidence of a treatment effect is seen when comparing the two treatment arms. While the clinical trial is proceeding, the principal investigators will not have access to interim data regarding overall treatment efficacy by study arm.

### Primary quantitative analysis approaches

Reflecting the cluster-randomized design of TEACH, the primary analysis of treatment effect on adherence will use assigned treatment as a predictor in a logistic mixed-effects model with each observation of adherence as the outcome [59], with appropriate random effect terms including center, patient (nested within center), and calendar day (again nested within center) to model between-observation covariance. Models will also include each center’s baseline pre-C-SAT/SBT-implementation data available during the 6-month baseline period; this substantially improves power to detect a treatment effect, as baseline adherence substantially varies between hospitals. Treatment effect significance will be assessed using a one-sided Wald-type test with type I error of 0.05. To assess robustness of the primary analysis and provide a prespecified “backup” analytic approach if there are tractability issues, a model excluding calendar day effect, and a model fit using generalized estimating equations, are prespecified supportive analytic approaches.

The co-primary ventilator-free days outcome will have a highly skewed distribution, due to a high proportion of deaths, and appreciable proportions of surviving patients with 0 ventilator-free days, or ventilator-free days at or near the maximum possible value of 27. The primary analysis approach for the ventilator-free day outcome is effectively an adjusted rank-based analysis, implementing a proportional odds model including center as a random effect [60], including baseline as well as intervention ventilator-free days as outcomes, and adjusting for covariates including patient age, Charlson Comorbidity Index, PaO_2_/FiO_2_ ratio, and COVID-19 diagnosis [61]. Fitting this model for the ventilator-free day outcome at a granularity level achieving satisfactory convergence (selected in a prespecified fashion independent of treatment effect), a likelihood ratio test with type I error of 0.05 will assess significance of the TEACH intervention by comparing deviance of the model with three levels of treatment (baseline, control, and TEACH) to the corresponding model with treatment modeled as baseline versus intervention only.

### Sample size estimate

Based on preliminary power analyses (see below), we plan a 36-month intervention period that includes a 90-day run-in period. Patients intubated during the run-in period will not be included in the final analysis. Estimated enrollment in the final analysis is up to 9900 mechanically ventilated patients over 33 months. Study duration and enrollment targets may be adjusted prior to the intervention period based on C-SAT/SBT adherence and other data observed during the pre-intervention baseline period.

### Type I error control and power analysis

It is questionable to attribute any observed reduction in ventilator-free days in the TEACH treatment arm to the intervention if a significant TEACH effect on adherence is not observed. Therefore, a formal hypothesis test of a TEACH treatment effect on the ventilator-free days outcome will be carried out, with type I error of 0.05, if and only if the TEACH effect on adherence is significant at the 0.05 level. This approach limits type I error to 0.05 for assessment of both outcomes.

### Adherence outcome power

For power estimation, we simulated intervention data using observed adherence proportions at each center during the baseline period, assuming fourfold improvement from baseline in relative adherence odds in the control arm in years 2–4, with higher improvement in the TEACH arm.

Study adherence data will exhibit clustering at the patient and hospital level and possibly by calendar day. We used a partially heuristic approach to power estimation, incorporating the variance inflation factor for clustered studies. Considering the intraclass correlation observed in the baseline study data and the distributions of eligible ICU days per study patient, a heuristic, conservative derivation yields a variance inflation factor of approximately 4.5 for adherence data [62] indicating effective sample size for power estimation can be estimated as actual number of eligible patient days divided by 4.5. Under these assumptions, and conservatively estimating sites’ patient volume for the three study years using 2019 levels incremented by 4% per year, estimated power to detect a significant TEACH effect on adherence is 86.4% if TEACH increases site adherence odds at least 1.333-fold versus control, exemplified by control-arm adherence increase from 40 to 72.7% versus TEACH-arm increase from 40 to 78.0%. Estimated power is 95.4% if the TEACH-related odds ratio is 1.4, exemplified by a site’s adherence increasing from 40 to 72.7% in the control arm versus 40 to 78.9% in the TEACH arm.

### Power for effectiveness outcomes

Power estimations simplistically assume the TEACH intervention will improve ventilator-free days among some percentage of surviving patients within an institution who would have been extubated within 28 days by a single day, compared to baseline-phase ventilator-free days at that same center. We assume no TEACH-related improvement in mortality rates or proportion of patients not extubated by day 27. At control sites, we assume no improvement in ventilator-free day distributions from baseline levels.

Simulations were carried out using final baseline data (with simulated post-intervention data obtained by resampling from each institution’s baseline cohort and then modifying ventilator-free days when appropriate due to modelled TEACH effect) and projected enrollment numbers for the 3 years of the intervention phase. Conservatively, a level of ventilator-free day “granularity” achieving satisfactory model convergence in effectively 100% of simulations was used. From these simulations, estimated power to detect a significant TEACH effect is approximately 87% if TEACH improves ventilator-free days by 1 day (compared to the usual care intervention) among two-thirds of surviving patients ventilator-free within 28 days of intubation. Estimated power is reduced to 51% if this one ventilator-free day improvement occurs among only one-half of such surviving patients.

### Early stopping rules and contingency plan

We will conduct two interim efficacy analyses approximately 12 and 24 months after trial launch to evaluate performance of TEACH intervention versus control. If introduction of the TEACH enhancement plus usual audit and feedback approach leads to substantially greater uptake and adherence during the first part of the study period versus control, such that the benefits of using TEACH far exceed the harms, the DSMB may recommend to stop the clinical trial early, and significant findings will be reported. These interim looks will use prespecified conservative O’Brien-Fleming [63] stopping boundaries, interim looks have only minor effects on effective study power, and early stopping occurs only if nominal statistical significance is substantial. Early stopping for futility is not warranted in our setting, as this trial will provide valuable information on various outcomes and facets of implementation even if adherence rates are ultimately not significantly different.

If the TEACH mechanism demonstrates significantly better adherence to C-SAT/SBT and increased ventilator-free days over the usual audit and feedback approach at an early interim analysis, leading to early stopping of the clinical trial, further actions would be deliberated by the principal investigators and sponsor, with the DSMB having an advisory role. As part of this deliberation, the study principal investigators may propose adjusting or redesigning the existing study, for example, eliminating the sustain measurement in later years and conducting a cost-effectiveness analysis to compare relative implementation performance costs of the TEACH method versus usual audit and feedback approach.

## Discussion

To our knowledge, TEACH will be the first clinical trial to compare a telehealth intervention augmenting a usual audit and feedback implementation versus a usual audit and feedback implementation alone in improving adherence to C-SAT/SBT in a prospective, multicenter type II hybrid effectiveness-implementation cluster-randomized setting. This trial builds on our prior collaborative work implementing computerized protocols for mechanical ventilation that drive adherence to evidence-based low tidal volume lung protective mechanical ventilation [50, 64], taking advantage of a tele-critical care program that includes physicians, advanced practice providers, respiratory therapists, nurses, and pharmacists to help facilitate and coordinate bedside nursing and respiratory care activities for mechanically ventilated patients.

Our study design has several strengths. First, we compare our combined strategy of telehealth real-time monitoring of patients eligible for C-SAT/SBT with usual audit and feedback to usual audit and feedback alone [65]. This design, which tests the incremental value of real-time telehealth oversight in addition to audit and feedback, is a recommended implementation science study design [66]. Second, Intermountain telehealth and tele-critical care programs are similar to telehealth systems in common use nationwide, aiding the generalizability of our findings. Third, this cluster-randomized clinical trial is being performed at hospitals sharing a common electronic medical record, standardized analgesia and sedation protocols, and computerized ventilator management protocols. This allows us to focus on C-SAT/SBT implementation and will maximize signal-to-noise ratio in our data. Fourth, we include clinician surveys to help understand how attitudes toward C-SAT/SBT have evolved over time with this implementation as compared to pre-intervention clinician attitudes [67].

We also note limitations. Optimally, the unit of cluster randomization would have been individual ICUs, which would have increased our number of clusters from 12 hospitals to 15 ICUs. All hospitals have a single ICU except for Intermountain Medical Center, whose 4 ICUs with shared respiratory therapy and nurse staffing necessitate randomizing that hospital as one cluster to prevent between-arm contamination. Also, although we have a moderate number of ICUs, all study ICUs are within a single health system, and the tested implementation strategies may not directly be applicable to ICUs in other health systems.

Of note, our study does have an additional sustainment aim which will allow us to determine how well these strategies influence clinician behavior when less resources are applied to the ICUs.

In summary, the TEACH clinical trial will advance the science of implementation by evaluating how telehealth services may augment a usual audit and feedback implementation of evidenced based C-SAT/SBT in mechanically ventilated patients.

### Supplementary Information

**Additional file 1.** SPIRIT 2013 checklist.

**Additional file 2.** CONSORT 2010 checklist.

**Additional file 3.** DSMB Charter, Version Dated February 23, 2022. Charter outlining the roles, responsibilities, practices, and procedures of the TEACH Trial Data and Safety Monitoring Board.

**Additional file 4.** Nurse job aid for coordinated SAT and SBT.

**Additional file 5.** Respiratory therapist job aid for coordinated SAT and SBT.

**Additional file 6.** Intermountain Pain, Agitation, and Delirium Guideline for Mechanically Ventilated Patients.

**Additional file 7.** Guidelines for daily tele-critical care operations in support of real time audit and feedback for the TEACH Study.

## Data Availability

In order to protect patient privacy and comply with relevant regulations, identified data are unavailable. Requests for deidentified data from qualified researchers with appropriate ethics board approvals and relevant data use agreements will be processed by the Intermountain Office of Research, officeofresearch@imail.org.

## Abbreviations

CAM-ICU: Confusion assessment method for the ICU
CPAP: Continuous positive airway pressure
C-SAT/SBT: Coordinated SAT and SBT
DCC: Data coordinating center
DSMB: Data safety and monitoring board
FiO_2_: Fraction of inspired oxygen
ICU: Intensive care unit
Intermountain: Intermountain Health
PaO_2_: Partial pressure of arterial oxygen
PEEP: Positive end-expiratory pressure
PS: Pressure support
REDCap: Research Electronic Data Capture
SAT: Spontaneous awakening trial
SBT: Spontaneous breathing trial
TEACH: *T*Elehealth-*e*nabled, real-time *a*udit and feedback for *c*linician ad*h*erence
