# Supplementary material for: Implementation of coordinated spontaneous awakening and breathing trials using telehealth-enabled, real-time audit and feedback for clinician adherence (TEACH): a type II hybrid effectiveness-implementation cluster-randomized trial
Source: Implement Sci. 2023 Sep 21;18:45. doi: 10.1186/s13012-023-01303-1 (PMC10515061; doi:10.1186/s13012-023-01303-1)
Supplement: Supplementary file 3 — Additional file 3. DSMB Charter, Version Dated February 23, 2022. Charter outlining the roles, responsibilities, practices, and procedures of the TEACH Trial Data and Safety Monitoring Board. [file 13012_2023_1303_MOESM3_ESM.docx]

1. **Introduction**

This Charter is for the Data Safety and Monitoring Board (DSMB) for the study *Implementation of Coordinated Spontaneous Awakening and Breathing Trials Using Telehealth-Enabled, Real-Time Audit and Feedback for Clinician Adherence: A Type II Hybrid Effectiveness-Implementation (TEACH study).*

The Charter is intended to be a living document. The DSMB may wish to review this Charter at regular intervals to determine whether any changes should be considered.

1. **Responsibilities of the DSMB**

The DSMB is responsible for safeguarding the interests of study participants, assessing the safety and efficacy of study procedures, ensuring data quality, evaluating clinical equipoise, and for monitoring the overall conduct of the study. In order to do this effectively in a trial, the DSMB is expected to review the data in an unmasked fashion in closed session.

The DSMB is an independent group advisory to the IRB and the investigators, and is required to provide recommendations about starting, continuing, and stopping the study. DSMB members must be cleared of any potential Conflict of Interest (COI) before participating in any meeting.

In addition, the DSMB is asked to make recommendations, as appropriate about:

- Efficacy of the study intervention (DSMB only)
- Benefit/risk ratio of procedures and participant burden
- Clinical equipoise (at onset and throughout the study)
- Ability of study design to answer the primary question
- Selection, recruitment, and retention of participants
- Adherence to protocol requirements
- Completeness, quality, and analysis of measurements
- Data and statistical analysis plan
- Initial protocol and data reporting shell
- Amendments to the study protocol, including whether any new data from other sources affect the equipoise of the study being monitored
- Impact of any changes in enrollment targets on the power of the study and whether ultimately the scientific questions can be adequately addressed
- Performance of individual centers
- Participant safety
- Notification of and referral for abnormal findings
- Review of the primary endpoint paper, methods and result sections
- Participant safety and parent study burden of proposed ancillary studies, including whether the total burden of ancillary studies might compromise the parent study

1. **Organization and Interactions**

The DSMB Chair will primarily communicate with the TEACH Study Contact Principal Investigator (PI), Colin Grissom, MD; and the Co-Principal Investigators Raj Srivastava, MD, MPH and Richard Holubkov, PhD. The Contact PI will ensure that the NHLBI is updated on communication between the DSMB and TEACH Study PIs. The TEACH Study PIs will communicate with the TEACH Study Coordinating Council to discuss or disseminate DSMB communication to other study investigators. The TEACH Study organization and key roles are listed below:

PIs

Colin Grissom MD

Raj Srivastava MD MPH

Rich Holubkov PhD

Coordinating Council

Colin Grissom, MD – Clinical Effectiveness Core

Raj Srivastava MD MPH – Implementation Science Core

Rich Holubkov PhD – Data and Statistics Core

Andrew J Knighton PhD CPA – Implementation Science

Doug Wolfe MBA – Implementation Operations

Lindsay Leither DO – Field Clinical Operations and Clinical Effectiveness Cores

Ithan Peltan, MD MSc – Clinical Effectiveness Core

Workstream Leaders

Vineela Maddukuri – Research Operations

Rich Holubkov PhD – Statistics/University Utah Statistical Data Center

Andrew J Knighton PhD CPA – Implementation Science

Doug Wolfe MBA – Implementation Operations

Lindsay Leither DO, Carrie Winberg RRT, Chris Jones RN – Field Clinical Operations

David Guidry MD – Telehealth Operations

Jason Jacobs PhD – Measurement and Reporting

David Edwards PhD, Pallavi Ranade-Kharkar PhD MS - Technology

Other Participants:

Sam Brown, MD MS – Clinical Effectiveness Core

Perry Gee PhD MSN – Clinical Effectiveness Core

Griffin Olsen MD – Implementation Science Core

Jacob Keane PhD – Implementation Science Core

Jay Berry MD MPH – Implementation Science Core

1. **DSMB Members**

DSMB members are listed below and in Appendix A with accompanying bio-sketches. The DSMB Executive Secretary (ES) will take minutes and work with the Chair to produce final recommendations and meeting report. Terri Clayson from Intermountain will serve as the ES and take minutes for the open session of DSMB meetings. She will prepare minutes for the meeting and send to the Chair, Anne Sales, who will add any additional minutes, reports, or recommendations from closed sessions of the DSMB. The DSMB Chair is responsible for assuring the accuracy and timely transmission of the final recommendations and DSMB’s report. *Ad hoc* members may be added to the DSMB to supplement expertise, as necessary for single or multiple meetings.

Data Safety Monitoring Board:

Anne Sales PhD RN – DSMB Chair, Implementation Science

Stephen Joffe MD MPH – Ethicist

Maria Mori Brooks PhD - Biostatistician

Damon Scales MD – Critical Care

Meeta Kerlin MD MSCE – Mechanical Ventilation

1. **Scheduling, Timing, and Organization of Meetings**

The purposes of the first meeting are to:

- Review this Charter;
- Provide an overview of TEACH Study activities; and
- Review the protocol and data reporting shell and make recommendations for changes related to human subjects’ safety and ethics.

The final study protocol will be distributed to the DSMB at least two weeks prior to the second DSMB meeting. The purpose of the second DSMB meeting will be to finalize and approve the study protocol.

Enrollment in a study cannot begin until the DSMB’s recommendation for approval has been accepted by the NHLBI Program Office, and IRB approval has been obtained.

Meetings are held approximately twice a year, with additional meetings scheduled as needed. The NHLBI Program Office will be notified of scheduled meetings in advance should Program Officer and/or delegate wish to monitor the meeting.

- Review of interim data analyses will occur annually for the first two years of the study. This will provide an interim data analysis at approximately 33% and 66% of study enrollment.
- Ad hoc meetings may be scheduled as needed

The agenda for DSMB meetings and calls may be drafted by the study biostatistician. The study biostatistician will finalize the agenda after consultation with the DSMB Chair. The agenda and meeting materials will be distributed by the PI for open meeting material and the blinded statistician for closed meeting material, two weeks before each meeting. The NHLBI Program Office may receive this material at the same time as DSMB members.

When the agenda is sent out, and again at the beginning of each meeting, the ES or Chair will ask all DSMB members to state whether they have developed any new conflicts of interest since the last DSMB meeting. If a new conflict is reported, the Chair and study staff will determine if the conflict limits the ability of the DSMB member to participate in the discussion. Examples of conflict of interest include significant collaborations in publications or other activities during the past few years, employee reporting relationships, or financial or intellectual stake in the research to be monitored.

The DSMB will review adverse event data, other safety data, quality and completeness of study data, and enrollment data at each meeting to ensure proper study conduct. It will also conduct an ongoing assessment of clinical equipoise in the study. Study personnel should provide any new literature particularly pertinent to the study, along with their recommendation as to whether it affects the study conduct or design. In the case of clinical trials, at intervals, as noted above, the DSMB will also review formal interim analyses of the primary end point. Based on an overall assessment of risk and review of the data, the Board will make recommendations, including whether the study should continue and/or be modified.

In addition to regular meetings, it may be necessary to convene the DSMB urgently on an *ad hoc* basis to discuss new data or other information that raises questions about equipoise, safety, or anything else in the study.

The expertise of the attending members should be appropriate for the agenda of the meeting. It’s expected that all DSMB members will attend every meeting, but, this may not always be possible. Therefore, the DSMB may establish a quorum for voting. A quorum is three members. The Board Chair and in most instances the Biostatistician, must be present at all meetings. All standing Monitoring Board members are voting members. The Board may also decide in advance whether *ad hoc* members can vote.

1. **Meeting Format**

DSMB meetings will be organized into open, closed, and executive sessions.

- During **open sessions**, information will be presented to the DSMB by the study investigators, with time for discussion.
- During **closed sessions**, the DSMB, required staff from the study (generally a study statistician), and the NHLBI program staff at the Chair’s discretion, will discuss confidential data from the study, including information on efficacy and safety by treatment arm. Select unblinded staff from the study may stay to explain the data reports. If DSMB does not receive unmasked data, the DSMB will discuss and decide during each meeting whether to remain masked to the treatment assignments. The unblinded study statistician should be able to provide treatment assignments immediately should the DSMB wish to be unmasked during a meeting. If the closed session occurs on a conference call, steps will be taken to ensure that only the appropriate participants are on the call, and to invite others to re-join the call only at the conclusion of the closed session.
- The DSMB may hold an **executive session** in which only the DSMB members, and NHLBI program staff at the Chair’s discretion, are present in order to discuss study issues independently.

Voting on recommendations will follow [Robert’s Rules of Order](http://www.robertsrules.com/)**.**

If the executive session occurs on a conference call, steps will be taken to ensure that only the appropriate participants are on the call, and to invite others to re-join the call only at the conclusion of the executive session.

At the conclusion of the closed and executive sessions, the participants may be re-convened so that the DSMB chair may provide a summary of the preliminary recommendations and provide an opportunity for participants to clarify the recommendations. The meeting is then adjourned.

1. **Expedited Safety Reporting**

Safety events requiring expedited reporting (as defined in DSM Plan) will be sent to the DSMB Chair, or designated members with expertise in critical care and mechanical ventilation and NHLBI program office within seven days of learning of the event. DSMB chair will respond to TEACH Study PIs and the NHLBI program office with recommendations within 14 days. Safety event definitions and reporting timelines are documented in the data and safety monitoring plan or protocol.

1. **Reports to the DSMB**

For each meeting, the study biostatistician, will prepare summary reports and tables to facilitate the oversight role of the DSMB. The DSMB will discuss at the first or subsequent meetings what data they wish to review and how it should be presented.

1. **Reports of DSMB Deliberations**

- Full Summary and Recommendations: The DSMB Chair, in conjunction with the ES, is responsible for the accuracy and transmission of DSMB meeting summary to the entire Board within 14 calendar days of the meeting. The summary must be signed by the DSMB Chair and will include reports of the closed session and executive session if an executive session was held. Study statistician may receive the full summary as decided by the DSMB chair on a meeting by meeting basis.
- Summary and Recommendations, edited so as not to expose the investigators to confidential or unblinded information and signed by the DSMB Chair, will be sent to the investigators within 14 calendar days after the meeting. These summary minutes and recommendations at minimum include a statement as to whether the study is approved to continue as planned, any requests for additional data, and response of the investigators to prior recommendations.

Requests for additional data from the investigators or DCC/statistician should include an expected due date.

DSMB recommendations must be submitted to the Intermountain Healthcare IRB.

- Action plan: Summary and Recommendations (including approval of revisions to the study protocol or statistical analysis plan), and the study’s follow-up plans will be submitted to the DSMB and NHLBI Program Office within 14 calendar days after the DSMB meeting.
- If the DSMB does not identify any safety or other protocol-related concerns, the Summary Report will state that:
- A review of outcome data, adverse events, and information relating to study

performance (e.g., data timeliness, completeness, and quality) across all centers took place on a given date

- No safety concerns were identified
- A review of recent literature relevant to the research took place,
- The study remains in clinical equipoise and
  - The DSMB recommended that the study continue without modification of the protocol or informed consent

1. **Statistical Monitoring Guidelines**

The DSMB will review the adequacy of the statistical monitoring plan. The final plan, whether part of a research protocol or separate document, will be maintained as an appendix to this Charter. The DSMB should discuss the statistical monitoring procedures that will be followed to guide recommendations about termination or continuation of the trial. These procedures could include guidelines for early termination for benefit, futility, and/or safety reasons.

The statistical monitoring procedures should ensure the development of clinical trial stopping rules for reasons of safety-related events or futility, which are clearly defined in advance as part of initial study review.

**DSMB Charter Approval**

The Charter was approved at the 2/23/202 meeting of the DSMB, as reflected in the minutes.

**Appendix:**

| **DSMB Members** | **DSMB Role** | **Role/Institution** | **Email** |
| --- | --- | --- | --- |
| Anne Sales PhD RN | DSMB Chair  Implementation Science | Professor, Department of Family and Community Medicine, Sinclair School of Nursing, University of Missouri | [asales@missouri.edu](mailto:asales@missouri.edu) |
| Steven Joffe MD MPH | Ethicist | Chief, Division of Medical Ethics, University of Pennsylvania | [joffes@upenn.edu](mailto:joffes@upenn.edu) |
| Maria Mori Brooks PhD | Biostatistician | Professor of Epidemiology and Biostatistics, University of Pittsburgh | [MBROOKS@pitt.edu](mailto:MBROOKS@pitt.edu) |
| Damon Scales MD | Critical care expert | Chief, Department of Critical Care Medicine,  Sunnybrook Health Sciences Centre  Professor, Interdepartmental Division of Critical Care and Department of Medicine, University of Toronto | damon.scales@sunnybrook.ca |
| Meeta Kerlin, MD, MSCE | Mechanical vent expert | Associate Professor of Medicine at the Hospital of the University of Pennsylvania  Associate Scholar, Center for Clinical Epidemiology and Biostatistics  Senior Fellow, Leonard Davis Institute of Health Economics Director, Acute care Outcomes and Health Services Research (AROHS) Group, Palliative and Advanced Illness Research (PAIR) Center | [MKerlin@pennmedicine.upenn.edu](mailto:MKerlin@pennmedicine.upenn.edu) |
