# Supplementary material for: Implementation of coordinated spontaneous awakening and breathing trials using telehealth-enabled, real-time audit and feedback for clinician adherence (TEACH): a type II hybrid effectiveness-implementation cluster-randomized trial
Source: Implement Sci. 2023 Sep 21;18:45. doi: 10.1186/s13012-023-01303-1 (PMC10515061; doi:10.1186/s13012-023-01303-1)
Supplement: Supplementary file 4 — Additional file 4. Nurse job aid for coordinated SAT and SBT. [file 13012_2023_1303_MOESM4_ESM.pdf]

Intermountain Healthcare

Coordinated SAT/SBT Implementation | RN Role

SAT/SBT – Updated Guidelines and Expectations

Spontaneous awakening and breathing trials (SAT/SBT) during invasive mechanical ventilation improve patient outcomes by decreasing delirium, VAPS, and length of stay in the ICU. Coordination between the RN/RT will ensure the highest degree of success possible for the patient.

Definition of terms and notes:

SAT – Spontaneous Awakening Trial: After completing the SAT safety screen process, the sedation is stopped for 30-120 minutes during which time the RN assesses and documents the patient's tolerance. If the patient is tolerating the absence of sedation it will not be turned back on. The RN should coordinate the SAT with the RT so that the SBT can be done during the time the patient is off sedating medications. To perform the SAT, turn off any sedative medications without weaning. Ensure there are adequate caregivers present at the bedside to reassure and attend to the patient as they awaken.

SBT – Spontaneous Breathing Trial: After completing the SBT safety screen process and coordinating with the RN to ensure proper timing, the SBT is done within 120 minutes after the sedation has been stopped. Note: It may take a little longer for some patients to be awake enough to complete the spontaneous breathing parameters. The parameters should be done without pressure support. An assessment of toleration should be documented along with measurements including MIF, VT, MV, and spontaneous RR. Average spontaneous tidal volume will be calculated by the charting of spontaneous parameters.

Changes to the process have been made:

The SAT/SBT trial requirement has been changed to a minimum of once per day. The goal is to complete this during the morning hours, if possible.

The SAT/SBT Algorithm is available and linked below. Additionally, a simplified algorithm or Quick Reference Guide has also been made available which focuses on the coordination between RT and RN. See links below.

Link to SAT/SBT Algorithm

Link to SAT/SBT Quick Reference Guide

Link to SAT/SBT resources on Critical Care Website

Documentation Is essential and will be audited:

Documentation of SAT is required each day on every ventilated patient using the "SAT Status" Field in the SAT/SBT band under Adult ICU Quick View. This field addresses the SAT safety screen/contraindications, the Pass or Fail of the SAT, and also if the patient does not need an SAT because they aren't sedated. The SBT charting will be done by the RT. It is visible in the RN charting so the information can be shared easily between the care team.

© 2022 Intermountain Healthcare. All rights reserved.

Tele Critical Care (TCC) may be supporting you in these efforts:

Expect that at some sites TCC may be calling to offer assistance with your ICU patients related to the SAT/SBT process. There may be tasks that TCC will offer to help with so that you can accomplish the SAT/SBT process with patients who are ready.

TCC may also call to ask about the SAT/SBT status for patients who are eligible to participate, and also to discuss questions, concerns and feedback about the SAT/SBT process. Please be proactive about working with TCC so that we can accomplish the SAT/SBT process for all the patients who are ready and able to participate.

Check your knowledge by reviewing the protocol:

Do you understand the relative contraindications for SAT?

Do you understand how to perform the SAT?

Do you know the failure criteria for the SAT?

Do you understand the SAT process?

Can you describe why it is important to coordinate the SAT/SBT process with the RT?

© 2022 Intermountain Healthcare. All rights reserved.
