# Supplementary material for: Implementation of coordinated spontaneous awakening and breathing trials using telehealth-enabled, real-time audit and feedback for clinician adherence (TEACH): a type II hybrid effectiveness-implementation cluster-randomized trial
Source: Implement Sci. 2023 Sep 21;18:45. doi: 10.1186/s13012-023-01303-1 (PMC10515061; doi:10.1186/s13012-023-01303-1)
Supplement: Supplementary file 5 — Additional file 5. Respiratory therapist job aid for coordinated SAT and SBT. [file 13012_2023_1303_MOESM5_ESM.pdf]

Respiratory Therapy SAT/SBT

SAT/SBT – Updated Guidelines and Expectations

Spontaneous awakening and breathing trials (SAT/SBT) during invasive mechanical ventilation improves patient outcomes by decreasing delirium, VAPS, and length of stay in the ICU. Coordination between the RN/RT will ensure the highest degree of success possible for the patient.

Definition of terms and notes:

SAT – Spontaneous Awakening Trial, after completing the safety screen process, the sedation is stopped for 30-120 minutes followed by assessment and documentation. If the patient is tolerating the absence of sedation it will not be turned back on. The RN should coordinate this SAT with the RT so that the SBT can be done during the time the patient is off sedating medications.

SBT – Spontaneous Breathing Trial, after completing the safety screen process and coordinating with the RN to ensure that SBT is done within 120 minutes after the sedation has been stopped. Note: It may take a little longer for some patients to be awake enough to complete the spontaneous breathing parameters. The parameters should be done without pressure support. An assessment of toleration should be documented along with measurements including VC, MIF, VT, MV, and spontaneous RR. Average spontaneous tidal volume will be calculated by the charting of spontaneous parameters.

Changes to the process have been made:

The SAT/SBT trial requirement has been changed to once per day. The goal is to complete this during the morning hours, if possible.

The SAT/SBT Algorithm is available and linked below. Additionally, a simplified algorithm or Quick Reference Guide has also been made available which focuses on the coordination between RT and RN. See links below.

Link to SAT/SBT Algorithm

Link to SAT/SBT Quick Reference Guide

Link to SAT/SBT resources on Critical Care Website

Documentation Is essential and will be audited:

Documentation is required using the spontaneous parameter fields, and SBT charting fields. The SAT charting will be done by the RN. It is visible in the RT charting so the RRT can see the results

When a patient cannot participate in an SAT/SBT trial documentation must include the reasons why. If documentation includes “not appropriate” a reason must also be specified.

TCC will be coordinating at designated sites:

Expect that at some sites TCC may be calling to offer assistance with your ICU patients related to the SAT/SBT process. There may be tasks that TCC will offer to help with so that you can accomplish the SAT/SBT process with patients who are ready.

© 2022 Intermountain Healthcare. All rights reserved.

TCC may also call to ask about the SAT/SBT status for patients who look ready to participate on the ABD dashboard. Please be proactive about working with TCC so that we can accomplish the SAT/SBT process for all the patients who are ready and able to participate.

Check your knowledge by reading the protocol:

Do you understand the relative contraindications for SBT?

Do you know the failure criteria for the SBT?

Do you understand the SBT process?

Do you understand the SBT Pre-Extubation Checklist?

Are you aware of the need to contact the physician for an order prior to extubation?

© 2022 Intermountain Healthcare. All rights reserved.
