# Supplementary material for: Implementation of coordinated spontaneous awakening and breathing trials using telehealth-enabled, real-time audit and feedback for clinician adherence (TEACH): a type II hybrid effectiveness-implementation cluster-randomized trial
Source: Implement Sci. 2023 Sep 21;18:45. doi: 10.1186/s13012-023-01303-1 (PMC10515061; doi:10.1186/s13012-023-01303-1)
Supplement: Supplementary file 6 — Additional file 6. Intermountain Pain, Agitation, and Delirium Guideline for Mechanically Ventilated Patients. [file 13012_2023_1303_MOESM6_ESM.pdf]

Intermountain Healthcare
Healing for life®

Pain, Agitation, and Delirium Guideline for Mechanically Ventilated Patients

Purpose: To provide standardized, evidence-based analgosedation for the prevention and management of pain, agitation, and delirium in critically ill adults within the ICU.

Not all mechanically ventilated patients need sedatives—analgesia alone may be adequate to reach RASS target.

Spontaneous awakening (SAT) and breathing trials (SBT) should be done according to protocol three times daily in most patients receiving continuous sedation (see SAT/SBT protocols). Try to eliminate medications with increased risk of delirium, and address sleep hygiene, eye glasses, hearing aids, comforting voices, music needs at least once per shift.

Set goal RASS (RASS Range: -5 to 5, usual goal is 0)

Pain

Assess pain using the appropriate pain scale: Is patient in pain? Any reason for possible pain?

- NO PAIN → At RASS goal?
  - YES → Reassess RASS/pain every 1-4 hours and perform SAT per protocol
  - NO →
    - **Patient too sedated (RASS lower than goal).** Perform SAT (hold sedation 30 minutes and reassess).
    - **Patient too agitated (RASS higher than goal).** Continue to Agitation and Delirium sections below
- YES → **Treat pain. Start analgesia with as needed dosing. Consider non-narcotics first.**
  Tylenol 650 mg PO/FT
  Oxycodone 5-10 mg PO/FT
  Fentanyl 25-50 mcg IV
  Hydromorphone 0.2-0.4 mg IV
  → Requiring >2 bolus doses/hour?
  - No → Reassess RASS/pain every 1-4 hours and perform SAT per protocol.
  - YES → **Consider adding **continuous IV analgesia infusion**
    Fentanyl IV infusion 25-150 mcg/hr
    Hydromorphone IV infusion 0.1-0.4 mg/hr
    †Morphine IV infusion 1-4 mg/hr
    → At RASS goal?

Agitation

Assess agitation. If sedation is necessary, consider additional bolus dosing of narcotics first, then start continuous IV infusion**
Propofol IV infusion 0-75 mcg/kg/min
Dexmedetomidine IV infusion 0-1.5 mcg/kg/hr
Ketamine 0-70 mcg/kg/min
Fentanyl IV infusion 25-150 mcg/hr
Consider additional bolus analgesia with ‖Fentanyl 25-50 mcg IVP prn agitation

→ At RASS goal?
- YES → Reassess RASS/pain every 1-4 hours and perform SAT per protocol.
- NO →
  - **Patient too sedated (RASS lower than goal).** Perform SAT (hold sedation 30 minutes and reassess).
  - **Patient too agitated (RASS higher than goal).** Continue to Delirium section

Delirium

Assess delirium.
CAM-ICU Positive?

- YES → **Non-Pharmacologic strategies**
  Eliminate offending medications. Encourage activity. Address sleep hygiene, eye glasses, hearing aids, comforting voices, and music

  ¶Pharmacologic Interventions
  Revisit pain/sedation management
  Melatonin 3-6 mg PO/FT qHS
  Trazodone 50-100 mg PO/FT qHS
  Olanzapine 5-10 mg PO q12h prn
  Quetiapine 25-50 mg PO/FT q6h prn
- NO → Continue current regimen, reassessing RASS/pain and CAM-ICU per unit protocol. Continue non-pharmacologic agitation/delirium prevention strategies. Agents for sleep, such as melatonin, may be used in non-delirious patients, or for delirium prevention

*Starting dosage ranges listed. Choose appropriate interval. Opioid tolerant patients may require higher doses.
**Start analgesics and sedatives at lowest, clinically appropriate dose and titrate by 50% to achieve target pain and/or sedation goals, respectively.
‖ If increased sedation needed, consider bolus dose fentanyl before increasing continuous IV infusion.
† Morphine should be avoided in patients with renal dysfunction. Morphine causes hypotension to a greater extent than fentanyl and hydromorphone.
¶Consider 12-lead ECG prior to initiating antipsychotics in high risk patients. Of note, no antipsychotic medication has shown consistent efficacy in reducing or treating delirium.

Approved by: Intermountain Healthcare Critical Care Operations, 3 March 2019
