# Supplementary material for: Implementation of coordinated spontaneous awakening and breathing trials using telehealth-enabled, real-time audit and feedback for clinician adherence (TEACH): a type II hybrid effectiveness-implementation cluster-randomized trial
Source: Implement Sci. 2023 Sep 21;18:45. doi: 10.1186/s13012-023-01303-1 (PMC10515061; doi:10.1186/s13012-023-01303-1)
Supplement: Supplementary file 7 — Additional file 7. Guidelines for daily tele-critical care operations in support of real time audit and feedback for the TEACH Study. [file 13012_2023_1303_MOESM7_ESM.docx]

**Additional File 7. Daily tele-critical care operations in support of the TEACH study.**

The tele-critical care operation will be staffed by 5-6 tele-critical care nurses and 1 tele-critical care respiratory therapist 24/7 that will monitor daily systemwide adherence to SAT and SBT, respectively, as part of their larger tele-critical care responsibilities.

Tele-critical care clinicians will intervene on an exception basis to help identify patients who are eligible for C-SAT/SBT but have not undergone one. Intervention using a “missed opportunity” model rather than systematic review of each patient daily is expected to improve efficiency and acceptability. Patient identification begins in the late morning after or near the end of rounds so that local ICU clinicians will have had an opportunity to adhere and document their results to the C-SAT/SBT protocol.

The TEACH intervention performed daily by tele-critical care clinicians will include five steps:

1. Identification: The tele-critical care clinicians will review the real-time electronic dashboard each morning to identify patients who have not had an SAT or SBT and may require one.
2. Evaluation: Between 11 AM and Noon each day, for each patient identified as potentially needing an SAT or SBT, the respective tele-critical care clinician will review charting in the electronic medical record to better understand each patient’s current medical situation, including existing physician orders or contra-indications. The tele-critical care clinician may also view the patient directly via a room camera to assess the patient’s daily status.
3. Outreach: The tele-critical care clinician will identify the name of the respective bedside nurse or respiratory therapist via the electronic medical record if the tele-critical care clinician determines that the patient is eligible for an SAT or SBT and it remains unclear why one has not been documented that day. The tele-critical care clinician will then attempt to contact the bedside clinician for the eligible patient via a wearable wireless communication device (Vocera™). If the bedside nurse or respiratory therapist does not answer, the tele-critical care staff member will leave a message prompting the nurse or respiratory therapist to ensure the SAT or SBT is completed and properly documented that day. If tele-critical care staff are unable to leave a message, they will try one more time that day to make contact.
4. Consultation: If the tele-critical care clinician is successful in speaking directly with the bedside clinician that day, the tele-critical care clinician will ask about the current status of the patient and the SAT or SBT. The tele-critical care clinician will then provide guidance or assistance as needed including (1) reviewing barriers or concerns to conducting the SAT or SBT; (2) educating on the C-SAT/SBT screening and performance care protocol; (3) stepping the bedside clinician through performance of an SAT or SBT with the current patient; and/or (4) educating on documentation requirements and assisting with documentation as needed.
5. Reporting: If contact is required, interaction information between the tele-critical care clinician and bedside clinician is documented in the real-time electronic dashboard. Periodic reports on patterns in TEACH interactions and feedback are provided to facility managers and local champions.
